# Bursting gamma oscillations in neural mass models

**DOI:** 10.3389/fncom.2024.1422159

**Published:** 2024-08-30

**Authors:** Manoj Kumar Nandi, Michele Valla, Matteo di Volo

**Affiliations:** ^1^Université Claude Bernard Lyon 1, Lyon, Rhône-Alpes, France; ^2^INSERM U1208 Institut Cellule Souche et Cerveau, Bron, France

**Keywords:** gamma oscillations, neural mass model, phase amplitude coupling, spiking neural network (SNN), synchronization, bursting

## Abstract

Gamma oscillations (30–120 Hz) in the brain are not periodic cycles, but they typically appear in short-time windows, often called oscillatory bursts. While the origin of this bursting phenomenon is still unclear, some recent studies hypothesize its origin in the external or endogenous noise of neural networks. We demonstrate that an exact neural mass model of excitatory and inhibitory quadratic-integrate and fire-spiking neurons theoretically predicts the emergence of a different regime of intrinsic bursting gamma (IBG) oscillations without any noise source, a phenomenon due to collective chaos. This regime is indeed observed in the direct simulation of spiking neurons, characterized by highly irregular spiking activity. IBG oscillations are distinguished by higher phase-amplitude coupling to slower theta oscillations concerning noise-induced bursting oscillations, thus indicating an increased capacity for information transfer between brain regions. We demonstrate that this phenomenon is present in both globally coupled and sparse networks of spiking neurons. These results propose a new mechanism for gamma oscillatory activity, suggesting deterministic collective chaos as a good candidate for the origin of gamma bursts.

## 1 Introduction

Oscillations are a hallmark of brain activity at the mesoscopic scale, thought to reflect the coherent dynamics of the underlying neural populations (Buzsaki, 2006). Oscillations have been recorded with different experimental techniques, such as Local Field Potentials, and are typically grouped in frequency bands, from slower delta and theta up to faster beta and gamma oscillations. Gamma oscillations (30–150 Hz) have received much attention because they have been associated with to cognitive functions and dysfunctions (Wang, 2010) and because they are thought to play a central role in the transfer of information between brain regions (Fries, 2015). In particular, narrow-band gamma oscillations, which typically cover the range of 30–80 Hz, have been found prominent during sensory stimulation (Ray and Maunsell, 2010) and cognitive processes such as attention (Fries et al., 2001; Bosman et al., 2012) and working memory (Pesaran and Shin, 2002). Moreover, gamma oscillations are particularly relevant in the Hippocampus, a brain region that plays a critical role in memory (Bird and Burgess, 2008). They are modulated by slower theta rhythms, an emergent interaction between frequency bands usually called Cross-Frequency Coupling (CFC) (Belluscio et al., 2012; Colgin, 2015; Bott et al., 2016). Such CFC is supposed to reflect an efficient transfer of information across spatial and temporal scales from an external source, responsible for slower theta oscillations, to the local computing circuit responding with gamma oscillations (Lisman and Jensen, 2013). In this study, we focus on the circuit mechanisms regulating such narrow-band gamma oscillations and their CFC with slower theta rhythms. This research study has been the subject of intense research in the last few decades. It has been exhibited that gamma oscillations can emerge in solely inhibitory networks thanks to synaptic delay or synaptic time scales (Brunel and Hakim, 1999) or in balanced sparse networks due to the constructive role of endogenous fluctuations (Di Volo and Torcini, 2018). Gamma oscillations are found in excitatory-inhibitory networks through the well-known Pyramidal Interneuronal Network Gamma (PING) mechanism (Tiesinga and Sejnowski, 2009). Here, the ping pong between an excitatory and inhibitory population is responsible for the emergence of gamma oscillations. These oscillations typically display a delay of a few milliseconds between excitatory population firing and the following inhibitory avalanche (Buzsáki and Wang, 2012).

A powerful method to investigate the emergence of oscillations is by employing neural mass or mean-field models. Neural mass models are low-dimensional descriptions of the population dynamics of spiking neural networks. They can be heuristic, like in the case of the pioneering study by Wilson and Cowan (1972) as well as derived directly from the details of the network model by di Volo et al. (2019) and Carlu et al. (2020). In the specific case of globally coupled quadratic integrate and fire-spiking neurons, an exact neural mass model has been obtained by Montbrió et al. (2015) based on the Ott-Antonsen ansatz (Ott and Antonsen, 2008). PING and Interneuronal Network Gamma (ING) oscillations have been observed in these models, reproducing with very good accuracy the population dynamics of the corresponding spiking neural network (Devalle et al., 2017, 2018; Segneri et al., 2020). In particular, PING oscillations are typically emerging because of a difference in the time scale regulating excitatory and inhibitory populations, which is responsible for the well-known delay between excitatory and inhibitory populations' spiking activity.

While these models allow us to determine basic mechanisms for the emergence of gamma oscillations, the features of these oscillations are quite different from what is usually observed in experimental recordings. In fact, recent studies depict that gamma oscillations appear in bursts, i.e., short high-amplitude oscillatory events separated by periods of low-amplitude oscillatory activity (Douchamps et al., 2024). The circuit origin and the function of these bursts are still unknown. It has been recently suggested that visually induced gamma bursts can be modeled through the noise on top of a damped harmonic oscillator (Spyropoulos et al., 2022). In the context of neural mass models, bursting oscillations (more specifically beta oscillations) have been modeled by settling the system close to a bifurcation from asynchronous to oscillatory activity and by adding external noise (Byrne et al., 2020; Kang et al., 2023). Similar study on gamma oscillations conveys the emergence of gamma bursts thanks to the presence of noise in the model (Tahvili and Destexhe, 2023). In this framework, oscillatory bursts' timing and features are random events related to external noise and are not an emergent property of the only neural network.

In this manuscript, we first consider globally coupled networks of excitatory and inhibitory quadratic integrate and fire neurons (Ermentrout and Kopell, 1986). The network includes variability in the inputs received by neurons (i.e., excitability), reflecting the natural heterogeneity observed in biological neural networks. We consider a Cauchy distribution for neuron excitabilities (i.e., the constant external current of neurons setting their isolated spontaneous activity), which allows us to derive the corresponding exact neural mass model (Montbrió et al., 2015). We first take advantage of the exact neural mass model to show that the network exhibits a very rich behavior when scanning the mean external drive to pyramidal neurons and the amount of heterogeneity in pyramidal neurons' excitability. By looking at network simulations, we observe asynchronous irregular regimes, the classic PING oscillatory regime (including a bistable regime of PING and asynchronous activity), as well as a different type of bursting gamma oscillations, emerging without the need for *ad-hoc* external noise. We found that gamma bursts in this region are due to deterministic chaos in the neural mass model. We called this type of burst intrinsic bursting gamma (IBG), to stress the difference with the classically used Noise-induced Bursts of Gamma (NiBG) oscillations. NiBG oscillations are observed in our model only by including the effect of finite-size fluctuations in the neural mass. Accordingly, they are not a deterministic emergent property of the neural mass model. Moreover, in direct network simulations of finite-size networks, the features of these oscillations strongly change with the number of neurons considered in the network, while the IBG oscillations are robust to changes in network sizes. In order to compare these two oscillations' types, we have estimated the PAC of the network to slower theta oscillations (10 Hz) in the two different regimes (IBG vs. NiBG). Interestingly, we found that the networks set in the vicinity of the IBG regime are characterized by higher CFC with theta oscillations than those networks demonstrating classical NiBG oscillations. This result demonstrates that deterministic IBG oscillations boost information transfer between regions and call for a reconsideration of the mechanisms at the basis of bursting gamma oscillations.

Finally, we confirm that these results can be generalized to sparse networks of excitatory and inhibitory neurons, as long as the number of connections is sufficiently large.

The manuscript is organized as follows: Methods are presented in Section 2, detailing the network model and its corresponding neural mass model. Additionally, we outline the methods employed to characterize model dynamics and analyze PAC. Section 3 presents the results of our study. Conclusion and discussions on our findings are presented in Section 4.

## 2 Methods

### 2.1 Network model

We consider a network consisting of N globally connected quadratic integrate-and-fire (QIF) neurons, with *N*^*E*^ being excitatory (E) and *N*^*I*^ being inhibitory (I) neurons. The membrane potential $v_{j}^{E}$ ($v_{j}^{I}$) of each excitatory (inhibitory) neuron j follows the subsequent differential equations:


(1) $$\begin{array}{lll} \tau_{m}^{E}{\dot{v}}_{j}^{E}\left(t\right) & = & {\left(v_{j}^{E}\left(t\right)\right)}^{2}+I_{j}^{E}+\xi_{j}^{E}\left(t\right)+2\tau_{m}^{E}S^{E}\left(t\right)+I_{0}^{\theta }\left(t\right)\\ \tau_{m}^{I}{\dot{v}}_{j}^{I}\left(t\right) & = & {\left(v_{j}^{I}\left(t\right)\right)}^{2}+I_{j}^{I}+\xi_{j}^{I}\left(t\right)+2\tau_{m}^{I}S^{I}\left(t\right), \end{array}$$

where, in Equation 1, $\tau_{m}^{X}$ with *X* = *E* (*I*) represents the excitatory (inhibitory) membrane time constant, and $I_{0}^{\theta }\left(t\right)=Asin\left(2\pi f_{\theta }t\right)$ represents an additional time-dependent external input. We fixed *f*_θ_ = 10 Hz and the amplitude *A* = 0 if not stated otherwise. $I_{j}^{E}$ ($I_{j}^{I}$) denotes quenched heterogeneity, which represents a constant input current that varies from neuron to neuron according to a Cauchy probability density function *P*(*I*^*X*^) centered at $I_{0}^{X}$ and with half-width at half-maximum (HWHM) Δ_*X*_ as shown in Equation 2.


(2) $$\begin{array}{l} P\left(I^{X}\right)=\frac{{\text{Δ}}_{X}}{{\left(I^{X}-I_{0}^{X}\right)}^{2}+{\text{Δ}}_{X}^{2}}\times \frac{1}{\pi }. \end{array}$$

Additionally, neurons receive independent noisy inputs. Specifically, the random variables ξ^*X*^(*t*) represent zero-centered Cauchy noise with a HWHM of Γ_*X*_. The choice of a Cauchy probability distribution allows us to exactly solve the equation for the neural mass model (Montbrió et al., 2015); see next section. The parameter Γ_*X*_ represents the amount of heterogeneity in neurons' external drive $I_{j}^{X}$, which can be seen as neurons' excitability, i.e., the distance from the threshold of firing. When Γ_*X*_ = 0, neurons are identical and follow the same differential equation with the same parameters. The external drive $I_{j}^{X}$ can change depending on the external input coming from other areas, but it is intrinsically heterogeneous between neurons. Such heterogeneity can be quantified by measuring the resting potential of cortical neurons (Di Volo and Destexhe, 2021), proving that the distribution of resting potential across neurons can be approximated to a Gaussian distribution with a standard deviation (SD) smaller than 1 (i.e., the corresponding Γ_*X*_).

Finally, if not stated otherwise, neurons interact with each other in an all-to-all manner through the mean post-synaptic activity. *S*^*X*^(*t*),


(3) $$\begin{array}{l} S^{E}\left(t\right)=\frac{1}{N_{E}}\overset{N_{E}}{\underset{j=1}{\sum }}J^{EE}\underset{j:t_{j}^{\left(n\right)}<t}{\sum }\delta \left(t-t_{j}^{\left(n\right)}\right)\\ \;-\frac{1}{N_{I}}\overset{N_{I}}{\underset{j=1}{\sum }}J^{EI}\underset{j:t_{j}^{\left(m\right)}<t}{\sum }\delta \left(t-t_{j}^{\left(m\right)}\right)\\ S^{I}\left(t\right)=\frac{1}{N_{E}}\overset{N_{E}}{\underset{j=1}{\sum }}J^{IE}\underset{j:t_{j}^{\left(n\right)}<t}{\sum }\delta \left(t-t_{j}^{\left(n\right)}\right)\\ \;-\frac{1}{N_{I}}\overset{N_{I}}{\underset{j=1}{\sum }}J^{II}\underset{j:t_{j}^{\left(m\right)}<t}{\sum }\delta \left(t-t_{j}^{\left(m\right)}\right), \end{array}$$

where *J*^*αβ*^ in Equation 3, is the synaptic coupling strength between post-synaptic neurons in population *α* and pre-synaptic neurons in population *β*. The post-synaptic potentials are *δ*-pulses, and the transmissions are instantaneous: $t_{j}^{\left(n\right)}$ denotes the n-th spike time emitted by the neuron *j*. When the membrane potential of the neuron reaches the threshold *v*_*p*_, it emits a spike, following which it is reset to *v*_*r*_. In the QIF model, *v*_*p*_ = −*v*_*r*_ = ∞. In numerical simulations, following a similar approach as outlined in Montbrió et al. (2015), the threshold and reset values have been approximated to *v*_*p*_ = −*v*_*r*_ = 100, and when neuron j reaches *v*_*j*_ ≥ 100, its voltage is reset to *v*_*j*_ = −100. Subsequently, the voltage remains constant at the reset value for a duration of $2\tau_{m}^{X}/100$. This accounts for the time required to transition from *v*_*j*_ = 100 to *v*_*j*_ = ∞ and from *v*_*j*_ = −∞ to *v*_*j*_ = −100. The spike emission of neuron j is recorded halfway through this period. If not stated otherwise, the system parameters are *J*^*EE*^ = 10.8, *J*^*IE*^ = 2.0, *J*^*EI*^ = 9.6286, *J*^*II*^ = 9.53939, and $\tau_{m}^{X}=5$ ms. We have used the fixed external current $I_{0}^{I}=2.0$ and the fixed population heterogeneity Δ_*I*_ = 0.10 and varied the excitatory external current $I_{0}^{E}$ and the population heterogeneity Δ_*E*_ (or noise amplitude Γ_*E*_). In the case of sparse networks, we randomly cut *P*_*C*_ = 80% of connections from each type of synaptic connectivity and rescale the synaptic strength by the following way: $J^{\alpha \beta }=J^{\alpha \beta }/\left(1.0-P_{C}\right)$ to keep the system equivalent (in its mean interaction strength) with the all-to-all network. The network dynamics are simulated using an Euler scheme with a time step of Δ*t* = 0.00015 ms. We discarded the initial transients lasting approximately *T*_*t*_≈10*s*. Time averages and fluctuations are typically calculated over time intervals of approximately *T*_*s*_ ≈ 100*s*. Our simulation involves networks consisting of *N* = 16000 with *N*^*E*^ = 8000 excitatory and *N*^*I*^ = 8000 inhibitory neurons. Notice that in the cortex, typically *N*^*E*^ ~ 4*N*^*I*^, but for the neural mass model, this does not change the emergent dynamics apart from finite-size fluctuations.

### 2.2 Neural mass model

If the network size is *N* → ∞, the spiking network dynamics for globally coupled neurons can be exactly reduced to a neural mass model (Montbrió et al., 2015; Clusella and Montbrió, 2022) using the reduction method initially developed for phase-coupled oscillators (Ott and Antonsen, 2008). This reduced model captures the system's behavior with only four collective variables: the mean membrane potential *V*^*X*^(*t*) and the instantaneous population rate *R*^*X*^(*t*). The neural mass model is described by the following equations:


(4a) $$\begin{array}{ll} {Ṙ}^{E}\left(t\right) & =\frac{2R^{E}\left(t\right)V^{E}\left(t\right)}{\tau_{m}^{E}}+\frac{{\text{Δ}}_{E}^{eff}}{{\left(\tau_{m}^{E}\right)}^{2}\pi }\\ {\dot{V}}^{E}\left(t\right) & =\frac{{\left(V^{E}\left(t\right)\right)}^{2}+I_{0}^{E}+I_{0}^{\theta }\left(t\right)}{\tau_{m}^{E}}-\tau_{m}^{E}{\left(\pi R^{E}\left(t\right)\right)}^{2} \end{array}$$

(4b) $$\begin{array}{l} +\left[J^{EE}R^{E}\left(t\right)-J^{EI}R^{I}\left(t\right)\right] \end{array}$$

(4c) $$\begin{array}{ll} {Ṙ}^{I}\left(t\right) & =\frac{2R^{I}\left(t\right)v^{I}\left(t\right)}{\tau_{m}^{I}}+\frac{{\text{Δ}}_{I}^{eff}}{{\left(\tau_{m}^{I}\right)}^{2}\pi } \end{array}$$

(4d) $$\begin{array}{ll} {\dot{V}}^{I}\left(t\right) & =\frac{{\left(V^{I}\left(t\right)\right)}^{2}+I_{0}^{I}}{\tau_{m}^{I}}-\tau_{m}^{I}{\left(\pi R^{I}\left(t\right)\right)}^{2}+\left[J^{IE}R^{E}\left(t\right)-J^{II}R^{I}\left(t\right)\right]. \end{array}$$

Here, ${\text{Δ}}_{X}^{eff}={\text{Δ}}_{X}+{\text{Γ}}_{X}$ is the effective level of disorder in the network, defined as the linear sum of noise amplitude Γ_*X*_ and the amount of heterogeneity Δ_*X*_. *R*^*X*^(*t*) is the mean firing rate, and *V*^*X*^(*t*) is the mean membrane potential of population *X* = *E* (*I*).

In order to account for finite-size fluctuations due to a finite number of neurons *N*, we have included in the neural mass model a Gaussian white noise ζ in the external current with the following properties: < ζ^*αβ*^(*t*)ζ^*α*^′*β*′(*t*′) >= *δ*(*t*−*t*′), when *α* = *α*′ and *β* = *β*′ and the noise correlation is zero otherwise (Vinci et al., 2023). Then the external current in Equations 4b, d becomes: $I_{0}^{\alpha }\left(t\right)=I_{0}^{\alpha }+\tau_{m}^{\alpha }\underset{\beta =E,I}{\sum }J^{\alpha \beta }\sqrt{\frac{R^{\beta }}{N^{\beta }dt}}\zeta^{\alpha \beta }\left(t\right)$ with *α* = *E* or *I*. Here *dt* is the integration step of the Euler scheme. and ζ^*αβ*^(*t*) is a random number between –0.5 and +0.5, taken from a normal distribution.

### 2.3 Measures of model dynamics and PAC

*Coefficient of variation (CV)*: To characterize the network dynamics, we measure the average membrane potential $V^{X}\left(t\right)=\overset{N^{X}}{\underset{i}{\sum }}v_{i}^{X}\left(t\right)/N^{X}$, the instantaneous firing rate *R*^*X*^(*t*), as well as the coefficient of variation (*cv*_*i*_) for each neuron *i*, calculated as the ratio of the SD σ_*i*_ to the mean (μ_*i*_) of the inter-spike intervals (ISIs) associated with the train of spikes emitted by the neuron *i*, $cv_{i}=\frac{\sigma_{i}}{\mu_{i}}$. The average CV of the population is defined as $CV=\underset{i}{\sum }cv_{i}/N$. Furthermore, to quantify the amplitude of oscillations in population activity, we have calculated the SD Σ_*V*_ of the mean membrane potential, $\Sigma_{V}=\sqrt{<{\left(V^{E}\right)}^{2}>-<V^{E}{>}^{2}}$.

*Power spectrogram*: we employed signal processing techniques to compute the frequency power spectrogram of the population activity. We consider the temporal sequence of the mean voltage *V*^*E*^(*t*), and we define the power spectrum as $S\left(t,f\right)=\left[{\hat{V}}^{E}\left(f\right)\right]{\left[{\hat{V}}^{E}\left(f\right)\right]}^{*}$, where ${\hat{V}}^{E}\left(f\right)$ is the Fourier transform of *V*^*E*^(*t*). We use the short-time Fourier transform (STFT) subroutine from the signal package of the *SciPy* library (Virtanen et al., 2020) to obtain the Fourier transform of *V*^*E*^(*t*) within a running time window of length Δ*T*_*win*_ at time t. Throughout this study, we perform STFT using 90% overlap, and the window length is Δ*T*_*win*_ = 0.05*s*. The spectrogram was visualized using a colormap, where the color code represents the normalized power spectral density *S*(*t, f*)/*max*(*S*(*t, f*)) obtained from the mean voltage *V*^*E*^(*t*) from the excitatory population. For better visualization, we use the log base 10 scale.

*Lyapunov exponent (LE)*: We estimate the LE {λ_*k*_} (Pikovsky and Politi, 2016) of the neural mass model described by Equation 4. The LE measures the average growth rates of small perturbations along the orthogonal manifolds. This is computed by linearizing the neural mass model as follows:


(5) $$\begin{array}{r} \tau_{m}^{E}\delta {Ṙ}^{E}=2[V^{E}\delta R^{E}+R^{E}\delta V^{E}]\\ \tau_{m}^{E}\delta {\dot{V}}^{E}=2V^{E}\delta V^{E}-2{\left(\pi \tau_{m}^{E}\right)}^{2}R^{E}\delta R^{E}+\tau_{m}^{E}[J^{EE}\delta R^{E}-J^{EI}\delta R^{I}[\\ \tau_{m}^{I}\delta {Ṙ}^{I}=2[V^{I}\delta R^{I}+R^{I}\delta V^{I}]\\ \tau_{m}^{I}\delta {\dot{V}}^{I}=2V^{I}\delta V^{I}-2{\left(\pi \tau_{m}^{I}\right)}^{2}R^{I}\delta R^{I}+\tau_{m}^{I}\left[J^{IE}\delta R^{E}-J^{II}\delta R^{I}\right]. \end{array}$$

The four LEs {λ_*k*_} with *k* = 1, ..., 4 can be obtained using the standard technique introduced by Benettin et al. (1980). A positive LE indicates chaotic behavior, while a negative exponent signifies stability. Using the Runge-Kutta 4th-order integration scheme with a time step of *dt* = 0.01*ms*, we calculate the LEs for the neural mass model. The integration was conducted for a duration of 100 s, after discarding a transient period of 10 s.

*Phase-amplitude coupling*: To understand how network dynamics is modulated by the phase of an external theta oscillatory signal, we introduce an external oscillatory input $I_{0}^{\theta }$ to both the neural mass model and the network simulation. This input is a periodic sinusoidal signal at a frequency of *f*_θ_ = 10 Hz. We use the PAC method to quantify the modulation of *V*^*E*^(*t*). PAC is defined as the modulation of the amplitude of the gamma component of the signal, *A*_*f*_*g*__(*t*), by the phase, *ϕ*_*f*_θ__(*t*), of the theta-frequency component. The initial step involves extracting the envelope of the gamma-frequency amplitude signal and the phase of the theta frequency signal. To do so, we use the *Hilbert* subroutine from the signal package of the *SciPy* library (Virtanen et al., 2020). Once we estimated the amplitude and the phase, we used the mean vector length (MVL) (Canolty et al., 2006) to compute PAC. This method estimates PAC from a signal of length M by associating the phase time series *ϕ*_*f*_θ__(*t*) and amplitude time series *A*_*f*_*g*__(*t*) with a complex-valued vector at each time point t. To assess the coupling between gamma *f*_*g*_ and theta *f*_θ_ frequencies, the MVL method calculates the magnitude of the average vector and determines PAC as follows (Canolty et al., 2006):


(6) $$\begin{array}{l} PAC=MVL\left(f_{g},f_{\theta }\right)=|\frac{1}{M}\overset{M}{\underset{t=1}{\sum }}A_{f_{g}}\left(t\right)e^{j\phi_{f_{\theta }}\left(t\right)}|. \end{array}$$

## 3 Results

### 3.1 Phase diagram and PING oscillations

**Figure 1:**
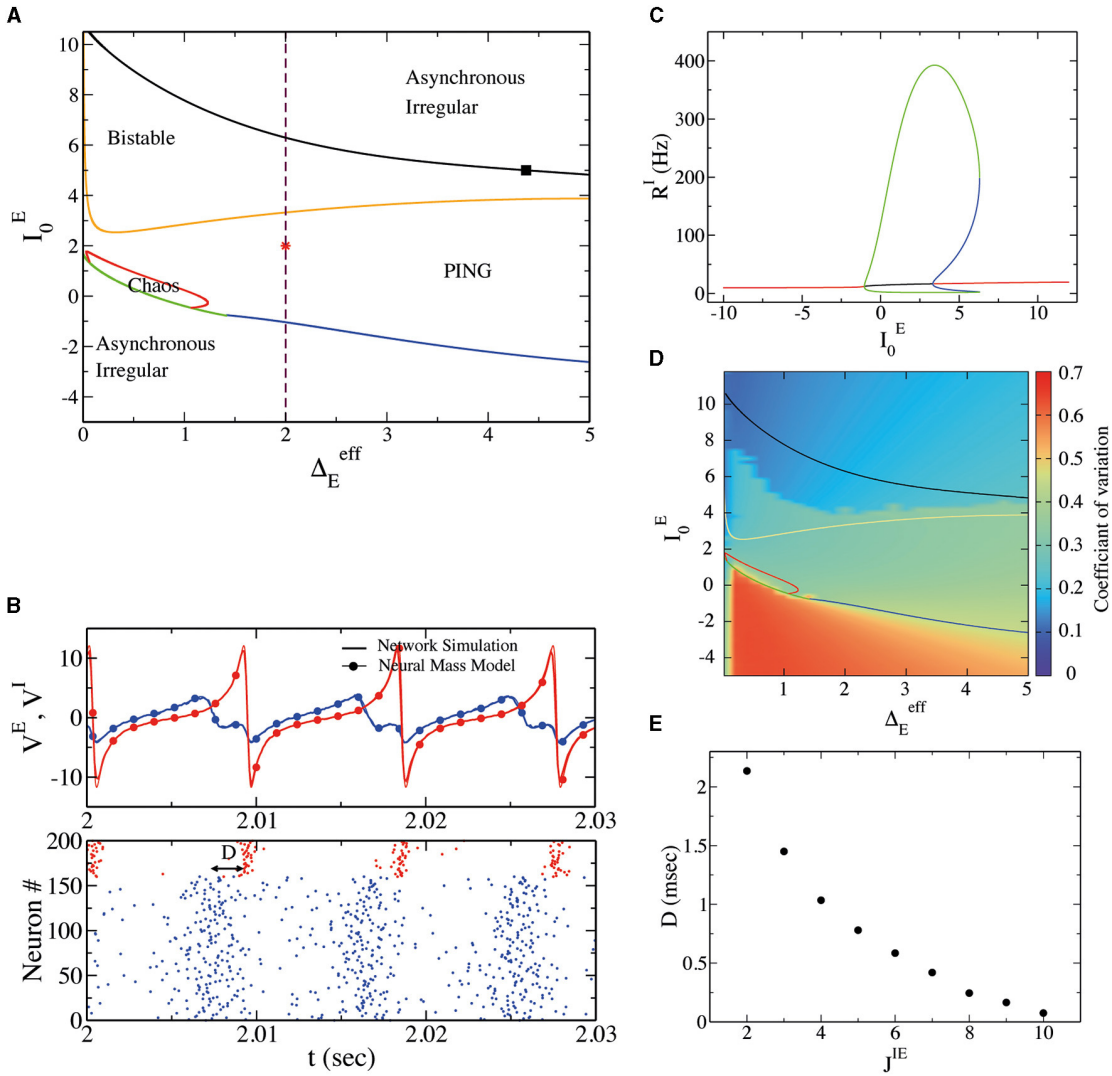
**(A)** Phase diagram for external drive $I_{0}^{E}$ and heterogeneity ${\text{Δ}}_{E}^{eff}$ obtained from the deterministic neural mass model. The blue line represents the supercritical Hopf bifurcation line separating a stable fixed point (asynchronous irregular in the network) from a stable limit cycle (PING oscillations in the network). The green line separates the stable fixed point and the chaotic region (IBG regime). The red line represents a period-doubling bifurcation and thus separates the chaotic region from the limit cycle region. The dark-yellow line represents a subcritical Hopf bifurcation, giving rise to the coexistence of asynchronous irregular dynamics and PING oscillations. Finally, the black line is a saddle-node of limit cycles separating the bistable region from a stable fixed point **(B)**. Top panel: average membrane potential for excitatory (inhibitory) neurons *V*^*E*^ (*V*^*I*^) in blue (red) from network simulations (continuous line) and neural mass model (line with filled circles) at the point indicated on the phase diagram as red star (${\text{Δ}}_{E}^{eff}=2.0$, $I_{0}^{E}=2.0$). Bottom panel: the corresponding raster plot for the excitatory and inhibitory neurons. D represents the delay in the synchronization of spike times between excitatory and inhibitory neurons. **(C)** The bifurcation diagram obtained with the software X-Windows PhasePlane plus Auto (XPPAUT) along the dashed line indicated in **(A)**. The red line represents stable fixed points, the green line is a stable limit cycle, and the blue line is an unstable limit cycle. **(D)** The CV of spiking neurons from the network simulation with respect to external drive $I_{0}^{E}$ and population heterogeneity Γ_*E*_ (in these simulations Δ_*E*_ = 0, so ${\text{Δ}}_{E}^{eff}={\text{Γ}}_{E}$). Notice that the CV depends on initial conditions (random in these simulations) in the bistable regime; that is why we observe a higher CV for oscillatory solutions and a lower CV for asynchronous solutions. **(E)** The delay D, at the same point indicated by the star in the phase diagram, concerns the variation of the synaptic strength between excitatory and inhibitory synapses, obtained from the neural mass model.

In order to investigate the dynamical regimes displayed by the spiking network, we performed an exploration of the phase space by employing the neural mass model and varying neurons' mean excitability $I_{0}^{E}$ and heterogeneity ${\text{Δ}}_{E}^{eff}$. Results can be observed in Figure 1A. At low $I_{0}^{E}$ we observe a stable fixed point in the neural mass model. This regime corresponds to asynchronous irregular dynamics in the spiking neural network. Neurons' irregularity can be measured by their (CV; see Section 2), reported in Figure 1D, close to *CV* = 0.7 in this regime. We have verified that the amplitude of oscillations Σ_*V*_ goes to zero as $\sqrt{N}$ in direct network simulations. We now focus on sufficiently large ${\text{Δ}}_{E}^{eff}$, say ${\text{Δ}}_{E}^{eff}>1.5$. As we can observe in Figure 1C, obtained for ${\text{Δ}}_{E}^{eff}=2$, by increasing $I_{0}^{E}$ we encounter a supercritical Hopf bifurcation (blue line in Figure 1A). This gives rise to a region of oscillations in population activity characterized by irregular neural activity (neurons' CV greater than zero), as can be observed in Figure 1D. In Figure 1B, we have reported the results of a numerical simulation for this regime. First, we observe a good agreement between numerical simulations of spiking neural networks and neural mass models. Then, we observe a delay *D* between the rise of excitatory neurons and inhibitory neurons activity of *D* ~ 2 ms, as in the classical PING-type oscillations. It is interesting to notice that the membrane time constant of excitatory neurons is here identical to that of inhibitory neurons. Our model thus suggests that a delay between pyramidal and interneuron bursts is a consequence of the structure of the model and not of neurons' or synaptic time constants. In order to test which structural parameter regulates such delay *D*, we have performed a numerical simulation by modifying the strength of synaptic coupling from excitatory to inhibitory neurons, namely *J*^*IE*^. In Figure 1E, we observe a decrease in *D* by increasing *J*^*IE*^, suggesting that the nature of the delay present in PING-type oscillations is due to the intensity of the network's connections.

More increasing $I_{0}^{E}$ (see Figures 1A, C), we encounter a subcritical Hopf bifurcation, giving rise to the coexistence of asynchronous irregular dynamics and PING oscillations. Eventually, a saddle-node of limit cycles is observed (black line in Figure 1A), and the PING oscillations become unstable. Above the black line in Figure 1A, the only stable solution is an asynchronous state.

**Figure 2:**
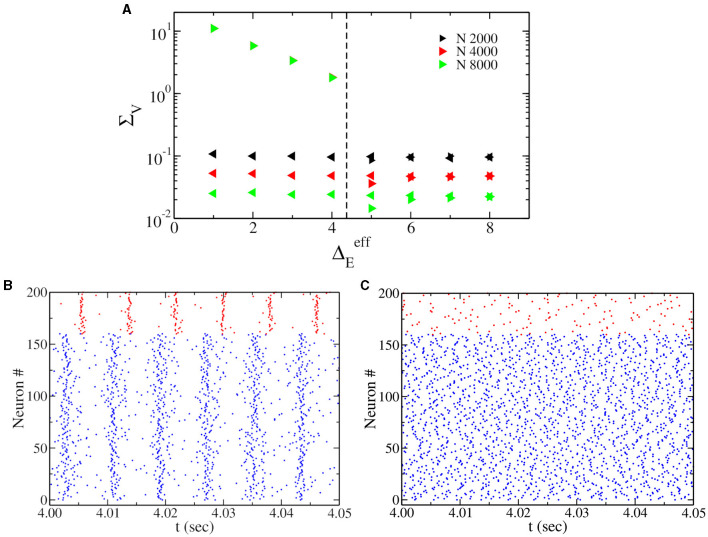
**(A)** Oscillations' amplitude Σ_*V*_, obtained by performing adiabatic simulations by first increasing (right triangles) and then decreasing (left triangles) ${\text{Δ}}_{E}^{eff}$ at $I_{0}^{E}=5.0$. Here Δ_*E*_ = 0, and we use Cauchy noise of amplitude ${\text{Γ}}_{E}={\text{Δ}}_{E}^{eff}$. Various network sizes *N* have been employed, as indicated in the legend. We observe that Σ_*V*_ goes to zero by increasing *N* in the asynchronous regimes and does not depend on *N* in the oscillatory regime. The dashed vertical line represents the saddle-node bifurcation point indicated by the black square in Figure 1A, obtained from the neural mass model. **(B, C)** Show the raster plot obtained for Γ_*E*_ = 2.0, indicating the coexistence of asynchronous and PING oscillations depending on the initial conditions.

We have performed hysteretic simulations in the neural network model to verify the existence of such a bistable regime, whose results are reported in Figure 2. We have performed this simulation for a fixed $I_{0}^{E}$, modifying the amount of noise amplitude ${\text{Δ}}_{E}^{eff}={\text{Γ}}_{E}$ (equivalent to heterogeneity in the neural mass model). This result affirms that in direct simulation, the network can oscillate (PING) or not, depending on the initial conditions. Moreover, the bifurcation point (saddle node of limit cycles) predicted by the neural mass model (dashed line in Figure 2A) corresponds very well to the value of ${\text{Δ}}_{E}^{eff}$, where the dynamics of the network become bistable.

Altogether, the richness of these oscillatory and asynchronous dynamics is somehow surprising given the simplicity of our model, which does not include synaptic dynamics or delays. Furthermore, we demonstrate that the CV of neurons is always larger than zero in direct numerical simulations of the spiking neural networks, indicating irregular spiking activity in the whole parameter space.

### 3.2 IBG oscillations

**Figure 3:**
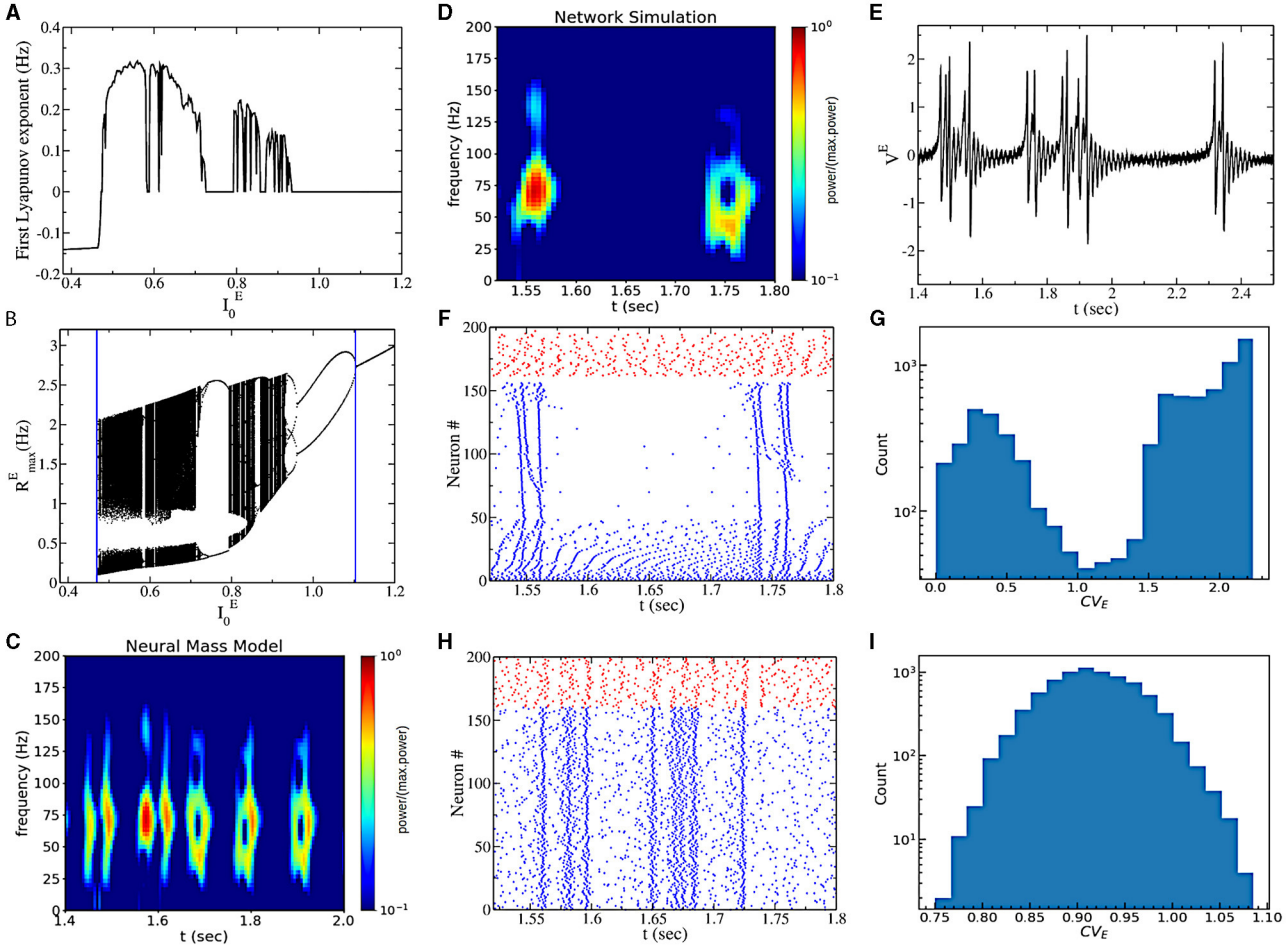
**(A)** The real part of the first Lyapunov exponent in the function of $I_{0}^{E}$. **(B)** Bifurcation diagram of the chaotic region generated by plotting the maximum value of the instantaneous firing rate *R*^*E*^ obtained from mean-field simulations. The blue vertical asymptote on the left is the limit point separating the asynchronous and the chaotic regions, while the vertical asymptote on the right is the point where the first period-doubling bifurcation appears. **(C)** A power spectrogram obtained from the neural mass model. **(D)** Power spectrogram of the mean voltage [presented in **(E)**]. **(E)** Time traces of the mean voltage of excitatory populations. **(F)** Raster plot of network simulations. We ordered the neurons according to their coefficient of variation, *cv*_*i*_. Blue dots represent the excitatory neurons, and red dots are inhibitory neurons. **(G)** Histogram of the coefficient of variation (CV) across neurons for the simulation of **(F)**. **(H)** Raster plot of network simulations for the same parameters as **(E)** but with Cauchy noise instead of heterogeneity. **(I)** CV for the simulation of **(H)**. In all panels, $I_{0}^{E}=0.5$ and ${\text{Δ}}_{eff}^{E}=0.4$. **(D–G)** are for heterogeneous networks (${\text{Δ}}_{eff}^{E}={\text{Δ}}_{E}=0.4$ and Γ_*E*_ = 0), and **(H, I)** are for networks with Cauchy noise (${\text{Δ}}_{eff}^{E}={\text{Γ}}_{E}=0.4$ and Δ_*E*_ = 0). The values of the power spectrogram < 10^−1^ are set to 10^−1^ for **(C, D)**.

The most interesting dynamical regime displayed by this model is observed for sufficiently low values of heterogeneity ${\text{Δ}}_{E}^{eff}$. This region is characterized by a positive LE (obtained from Equation 5) of the neural mass model (see Figure 3A), indicating sensitivity to initial conditions. As a strong indication of chaotic dynamics in the neural mass model, we indicate this regime as collective chaos, like in other previous studies (Nakagawa and Kuramoto, 1993; Olmi et al., 2010; Bi et al., 2021). Starting from a very small $I_{0}^{E}$, by increasing $I_{0}^{E}$, the real part of the maximum Lyapunov exponent λ encounters a discontinuous transition from negative to positive values (see Figure 3A). On the opposite side (for high drive $I_{0}^{E}$), we observe a transition from positive to zero values, as expected for a period-doubling cascade. Indeed, by reporting the Feigenbaum diagram (Figure 3B), we observe that chaos is initiated at high external drive $I_{0}^{E}$, through a period-doubling cascade. On the other side, for sufficiently small $I_{0}^{E}$ chaos is initiated through a discontinuous transition from a fixed point to chaotic dynamics, covering almost all the firing rate values in the confined range (from 0.1 to 2–3 Hz).

A closer look at the chaotic dynamics observable for low external drives $I_{0}^{E}$ reveals the presence of non-periodic bursting gamma oscillations in the neural mass model (see Figure 3C). We indeed observe periods of high-amplitude oscillatory activity (50–70 Hz) and periods of almost asynchronous (low-amplitude) activity of irregular duration. The bursting oscillatory periods last for approximately 100 ms. We call this regime intrinsic bursting gamma oscillations (IBG).

In order to understand the underlying neural mechanisms, we performed direct numerical simulations of the spiking neural network. We first consider the case with quenched heterogeneity of neurons' excitability (i.e., ${\text{Δ}}_{E}^{eff}={\text{Δ}}_{E}$, Γ_*E*_ = 0). We observe very similar dynamics to the ones predicted by the neural mass model (see Figures 3D, E). Furthermore, we observe that gamma bursts are elicited by a subgroup of bursting neurons. These neurons are active (almost) only during the gamma burst. This can be observed by looking at the raster plot of Figure 3F, where we ordered excitatory neurons according to neurons' coefficients of variation (*cv*_*i*_). Excitatory neurons with high *cv* activate during the burst, and neurons with lower *cv* are responsible for the background activity. We indeed observe a bimodal distribution of neurons' CV (Figure 3G). We then considered the case with Cauchy noise (i.e., ${\text{Δ}}_{E}^{eff}={\text{Γ}}_{E}$, Δ_*E*_ = 0). Interestingly, the population dynamics are the same (being described by the same neural mass model) as the model with no noise and heterogeneous excitabilities. Nevertheless, the temporal structure of the spiking activity of neurons is different. Now all neurons have similar firing statistics with high CV, and they all participate in the bursting event (Figures 3H, I).

The emergence of the IBG regime is dependent on the coupling structure of the model. We have performed numerical simulations (data not shown) indicating that an intermediate amount of coupling from excitatory to inhibitory neurons (*J*_*IE*_) is necessary for the emergence of IBG. For large values of *J*_*IE*_, the network displays asynchronous activity with low firing rates, while for small values of *J*_*IE*_ the network displays asynchronous activity with large firing rates. A complete analysis of the role of different parameters can be an interesting direction for future studies to unveil the role of the coupling structure in the emergence of IBG.

### 3.3 Finite-size bursting gammas

**Figure 4:**
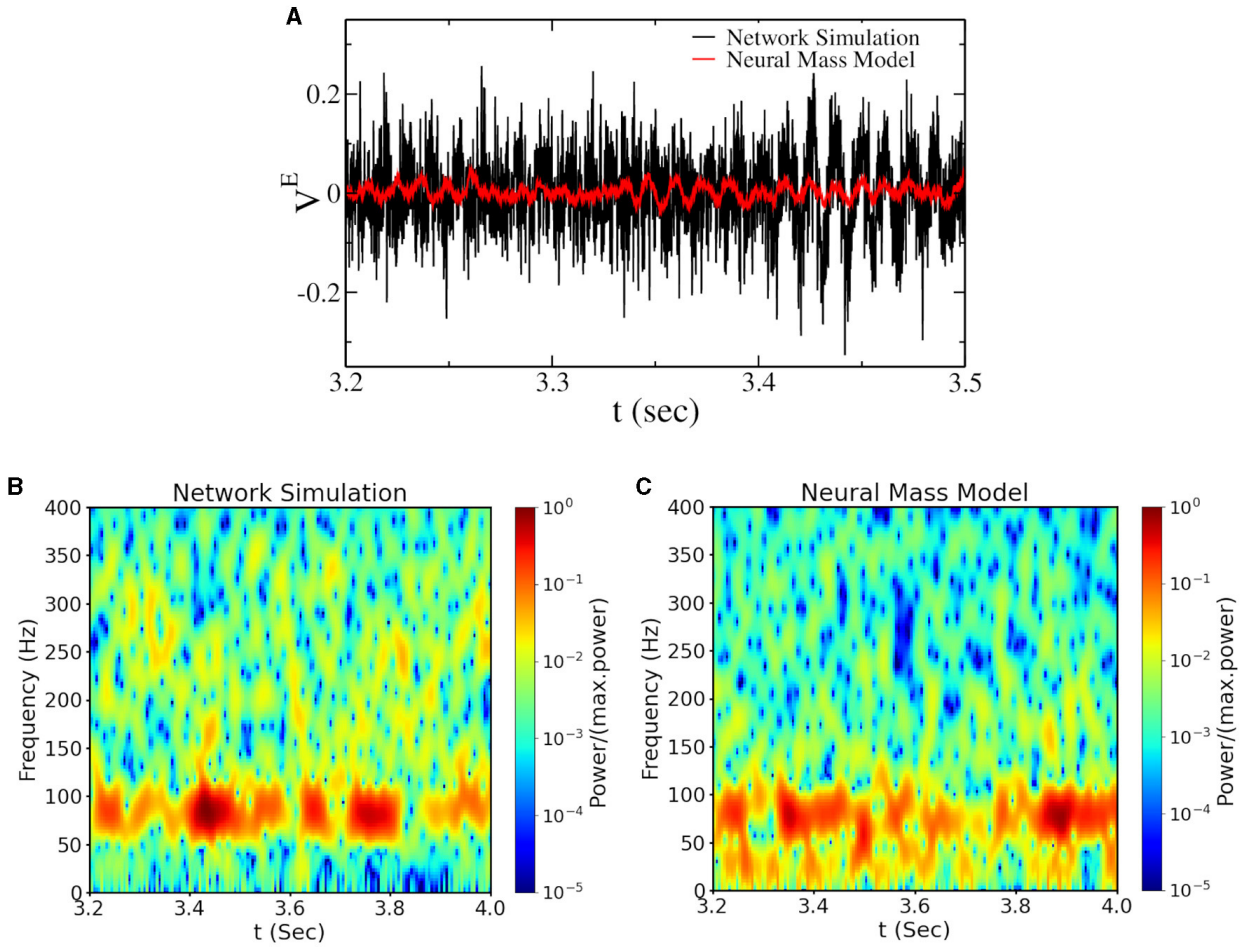
**(A)** Time traces of the mean voltage of excitatory neurons ($I_{0}^{E}=-3.0,{\text{Γ}}_{E}={\text{Δ}}_{E}^{eff}=3.0$) for a network of *N* = 16, 000 neurons—close to the Hopf bifurcation line (see Figure 1A). The black line represents the voltage from the network simulation, and the red line represents the same from the neural mass model with additive Gaussian noise (see Section 2). The spectrogram of the corresponding signal from network simulation and the neural mass model are plotted in **(B, C)**. The values of the power spectrogram < 10^−5^ are set to 10^−5^ for **(B, C)**.

Bursting gamma oscillations can be observed in spiking network simulations and also in the asynchronous region at large ${\text{Δ}}_{E}^{eff}$ if we set parameters sufficiently close to the Hopf bifurcation [see Figures 4A, B (black line in Figure 4A)]. In this regime, the power and the amount of gamma bursts depend on the network size, indicating that this is a finite-size effect that is supposed to vanish in the thermodynamic limit. To understand the origin of these gamma bursts, we have introduced finite-size fluctuations in the neural mass model, approximating finite-size fluctuations as white noise (Mattia and Del Giudice, 2002). With this approximation, we could reproduce gamma bursts as observed in network simulations (see the red line in Figure 4A and the power spectrogram in Figures 4B, C), thus confirming that they are due to finite-size fluctuations kicking the system up and down the Hopf bifurcation point. This represents the classical model for bursting oscillations due to external noise in neural mass models. We call this regime Noise-induced Bursting Gamma oscillations (NiBG). This regime is not an emergent property of the neural mass model, and the appearance of gamma bursts is governed by random fluctuation, which appears to us as a non-physiological mechanism. Nevertheless, it is still a possible mechanism in finite-size networks largely employed in the field. In the next section, we will compare the features of the NiBG and the deterministic IBG in terms of CFC with slower oscillations.

### 3.4 Cross-frequency coupling

**Figure 5:**
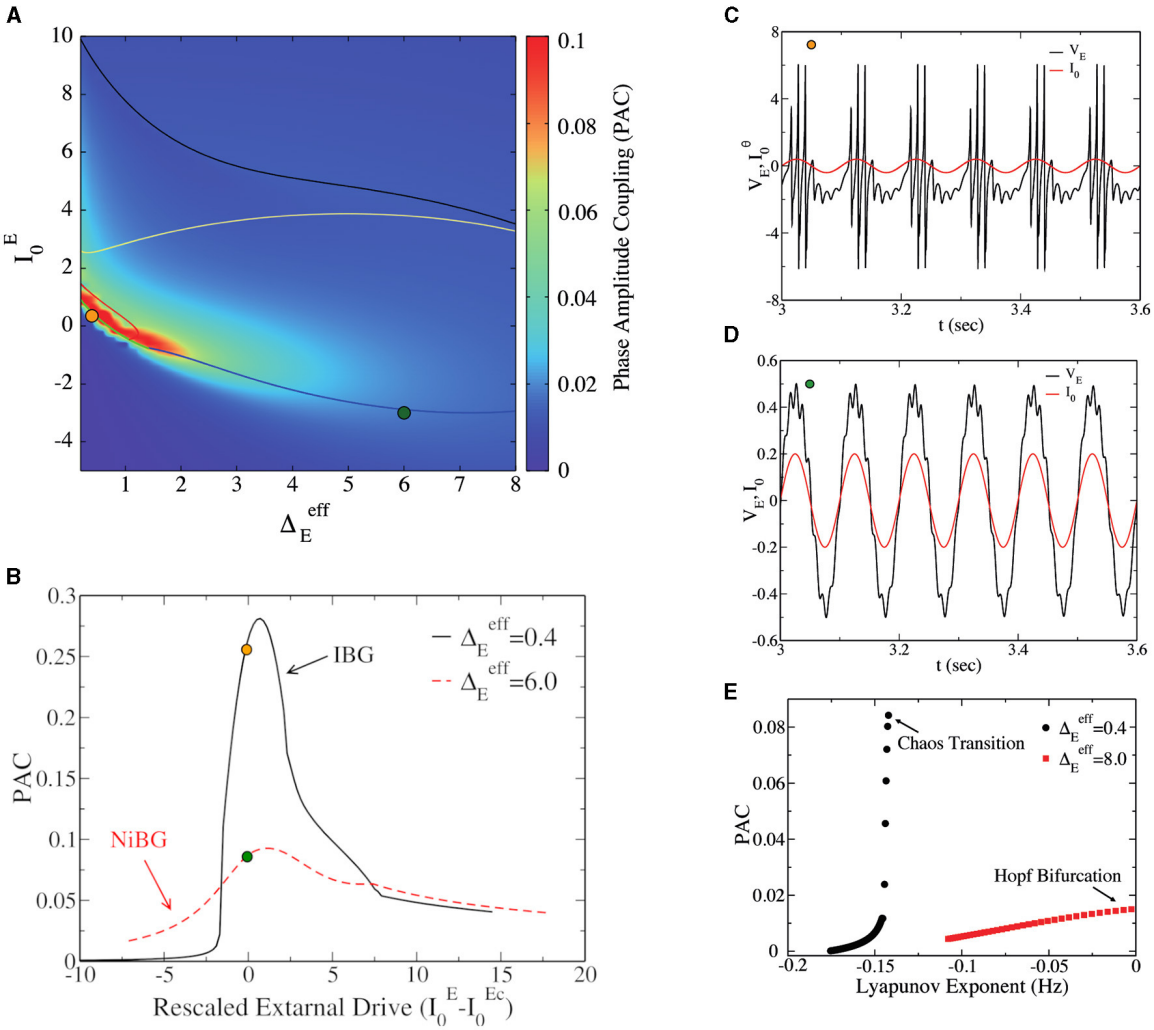
**(A)** Phase-amplitude coupling (PAC) to the external θ forcing of amplitude *A* = 0.04 vs. the external drive $I_{0}^{E}$ and the heterogeneity ${\text{Δ}}_{E}^{eff}$. This is a heat map of Figure 1A in the parameter space, and the IBG is inside the red circle lines around the orange dot, as in Figure 1A. **(B)** PAC value vs. the distance to the critical value of the external drive ($I_{0}^{E}-I_{0}^{Ec}$) at the two different population heterogeneities, ${\text{Δ}}_{E}^{eff}=0.4$ (black line) and ${\text{Δ}}_{E}^{eff}=6.0$ (red line). For the Hopf bifurcation (${\text{Δ}}_{E}^{eff}=6.0$), $I_{0}^{Ec}=-2.88$, while for the bifurcation to chaos (${\text{Δ}}_{E}^{eff}=0.4$), $I_{0}^{Ec}=0.47$. **(C)** Time traces of the mean excitatory voltage (black line) were obtained at the point represented by a dark-yellow circle in **(A)** (${\text{Δ}}_{E}^{eff}=0.4$, $I_{0}^{E}=0.35$). The red line represents the external theta drive. **(D)** The time traces of mean excitatory voltage and external theta drive at the state point are represented by the dark green circle (${\text{Δ}}_{E}^{eff}=6.0$, $I_{0}^{E}=-3.0$). **(E)** PAC vs. the real part of the maximum of Lyapunov exponent λ for different values of $I_{0}^{E}$ at two values of ${\text{Δ}}_{E}^{eff}$ (${\text{Δ}}_{E}^{eff}=0.4$ and ${\text{Δ}}_{E}^{eff}=8.0$). Data were obtained from the neural mass model. The amplitude of the external θ forcing signal is *A* = 0.04 for **(A, E)** and the amplitude is *A* = 0.2 for **(B–D)**.

What is the difference between the two types of bursting gamma oscillations (IBG vs. NiBG) shown in previous sections? A natural way to address this question is by considering the capacity of the network to respond to external stimulation or to slower oscillations. PAC between theta and gamma oscillations is known to be a measure of the capacity of the network to transfer information from one upstream region, oscillating at a theta frequency (10 Hz), to the other region, locally oscillating at a faster gamma frequency. PAC is present when the amplitude of gamma oscillations is modulated by the phase of the incoming theta oscillatory signal. A stronger PAC implies more efficient potential information transfer from one region to the local gamma circuit. In Figure 5A, we report the PAC (obtained using Equation 6) of the network as a function of $I_{0}^{E}$ and ${\text{Δ}}_{E}^{eff}$. We observe that PAC is very large inside and among the neighbors of the IBG regime. Notice that PAC is large, also outside the IBG regime but still close to it. In order to study the PAC close to the transition point from asynchronous dynamics to bursting oscillations, we report in Figure 5B the PAC of the network in function of the distance to the critical point $I_{0}^{E}-I_{0}^{E_{c}}$, where $I_{0}^{E_{c}}$ represents the transition from asynchronous to IBG regime. For the sake of comparison, we also report the PAC of the network close to the bifurcation point for PING oscillations in the NiBG regime. Choosing these two different values of ${\text{Δ}}_{E}^{eff}$ allows us to directly compare the PAC in IBG (observable at low ${\text{Δ}}_{E}^{eff}$) vs. NiBG (observable at high ${\text{Δ}}_{E}^{eff}$). We observe that PAC is maximum at the critical point for both cases, but it is much higher close to the IBG regime. As we can observe in Figures 5C, D, gamma oscillations have a much higher amplitude at the theta peak in the asynchronous regime at the fringe of the IBG regime.

The capacity of the network to couple to a slow theta cycle can be linked to the ability of the network to respond to external perturbations. In particular, the increased responsiveness of the network to external stimuli has been linked to its stability, measured via the amplitude of the LE λ in the asynchronous regime (Di Volo and Destexhe, 2021). In Figure 5E, we consider the relation between PAC and the real part of the maximum LE λ (as in Figure 3A) for different values of $I_{0}^{E}$ and two values of ${\text{Δ}}_{E}^{eff}$. We observe that PAC increases with λ, but the increase is much steeper for those asynchronous regimes in the vicinity of the IBG regime, i.e., close to the transition to chaos. The asynchronous regimes close to the Hopf bifurcation and PING oscillations have a much lower PAC. This result suggests that the stability of the asynchronous regime is important for responsiveness and the PAC, but that also this depends on the dynamical regimes in the neighborhood. Indeed, an optimal PAC appears in the surroundings of the IBG regime.

### 3.5 Sparse networks

**Figure 6:**
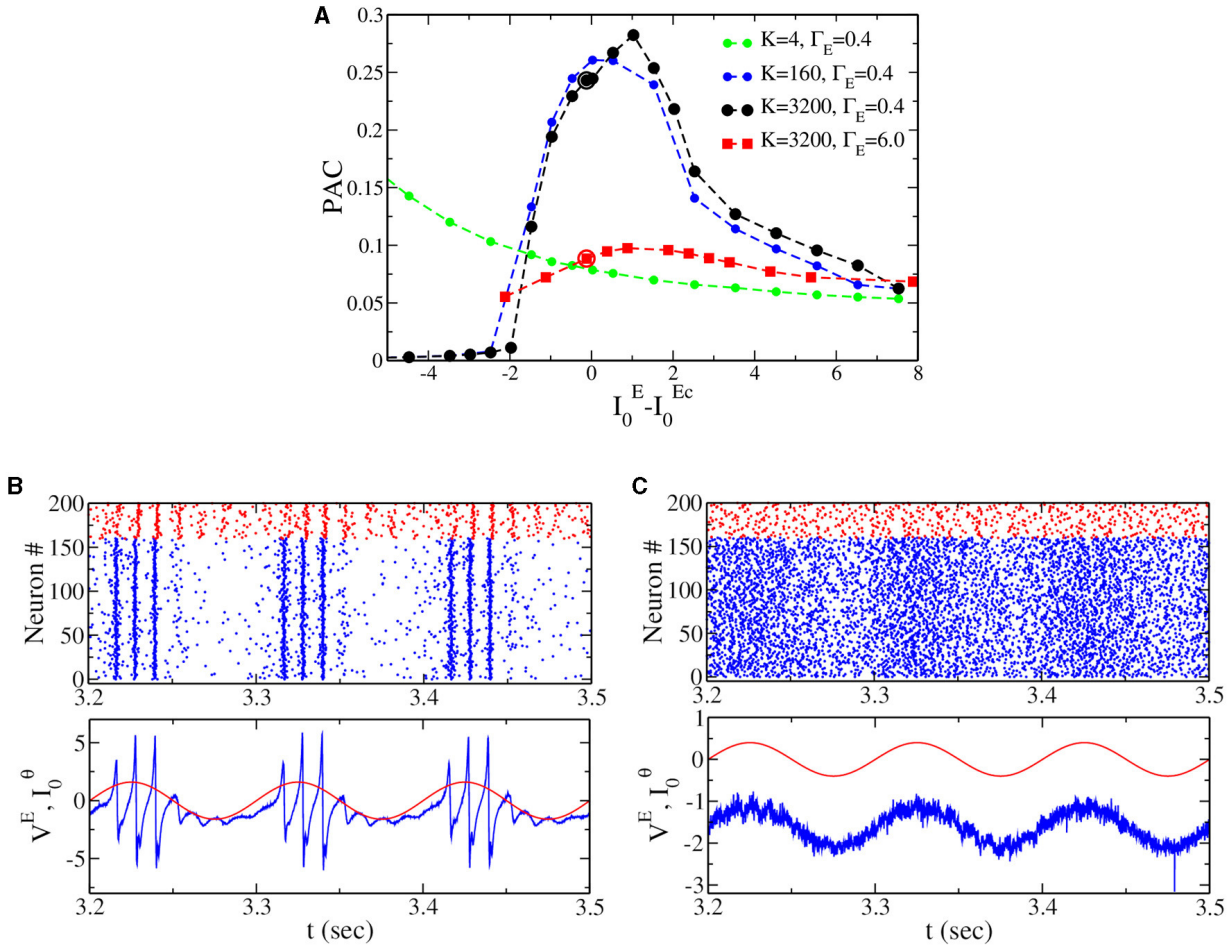
**(A)** Phase-amplitude coupling (PAC) to the external θ forcing of amplitude *A* = 0.2 vs. the distance to the critical value of the external drive ($I_{0}^{E}-I_{0}^{Ec}$) at two different population heterogeneity levels for sparse networks (same values as in Figure 5B, Γ_*E*_ = 0.4 and Γ_*E*_ = 6.0). The dashed line with solid circles represents the PAC values for different in-degree *K*. **(B)** Top panel: raster plot from network simulation [see black circle in **(A)**, Γ_*E*_ = 0.4, $I_{0}^{E}=0.35$]. Bottom panel: the corresponding time traces for the mean membrane potential for the excitatory population (blue) and the external theta drive $I_{0}^{\theta }$ (red). **(C)** The same as for **(B)**, but at the point indicated by the red circle in **(A)** (Γ_*E*_ = 6.0, $I_{0}^{E}=-3.0$).

All the results presented in the previous section have been obtained for globally coupled networks, mainly because this allows us to obtain the exact neural mass model description. In the following, we confirm that our result on the PAC in the IBG (i.e., Figure 5B) can be generalized to sparse networks. In order to do this, we randomly cut a percentage *P*_*C*_ of the connections, which gives a mean in-degree *K* = (1−*P*_*C*_)*N*, where *N* = 16, 000 is the network size. Notice that we properly rescale synaptic coupling to compare with the neural mass; see the Section 2 for details. Results are reported in Figure 6A. First, we observe (see dotted lines of Figure 6A) that for sufficiently large *K*, the dependence of PAC on the model's parameters is very similar to the globally coupled case (see the black line in Figure 5B). Nevertheless, when the mean in-degree *K* is very small (*K* = 4 in the figure), the shape of the curve changes, showing that when the network is too sparse, we do not observe an optimal PAC close to the IBG regime. Second, for sufficiently large *K*, we can reproduce the result obtained in Figure 5B for the neural mass model. In fact, we observe that the PAC is much larger close to the IBG region (black dotted line) at low ${\text{Δ}}_{E}^{eff}$ than the PAC close to the NiBG region (red squared line) at high ${\text{Δ}}_{E}^{eff}$. Gamma oscillations are indeed more or less equally distributed in the theta phase close to the Hopf bifurcation (see Figure 6C), while they have a much stronger amplitude at zero phase in the case of IBG oscillations (see Figure 6B). This result proves that the neural mass model can perform valuable predictions beyond its limits of applicability, as it has been discussed in previous studies in the context of networks with spike-frequency adaptation (Gast et al., 2020). It is important to notice at the same time that the structure of the network can play a crucial role in shaping the dynamics and the stability of neuronal networks, as previously shown (Di Volo and Torcini, 2018; Harris et al., 2023).

### 3.6 Frequency of oscillations vs. the model's parameters

**Figure 7:**
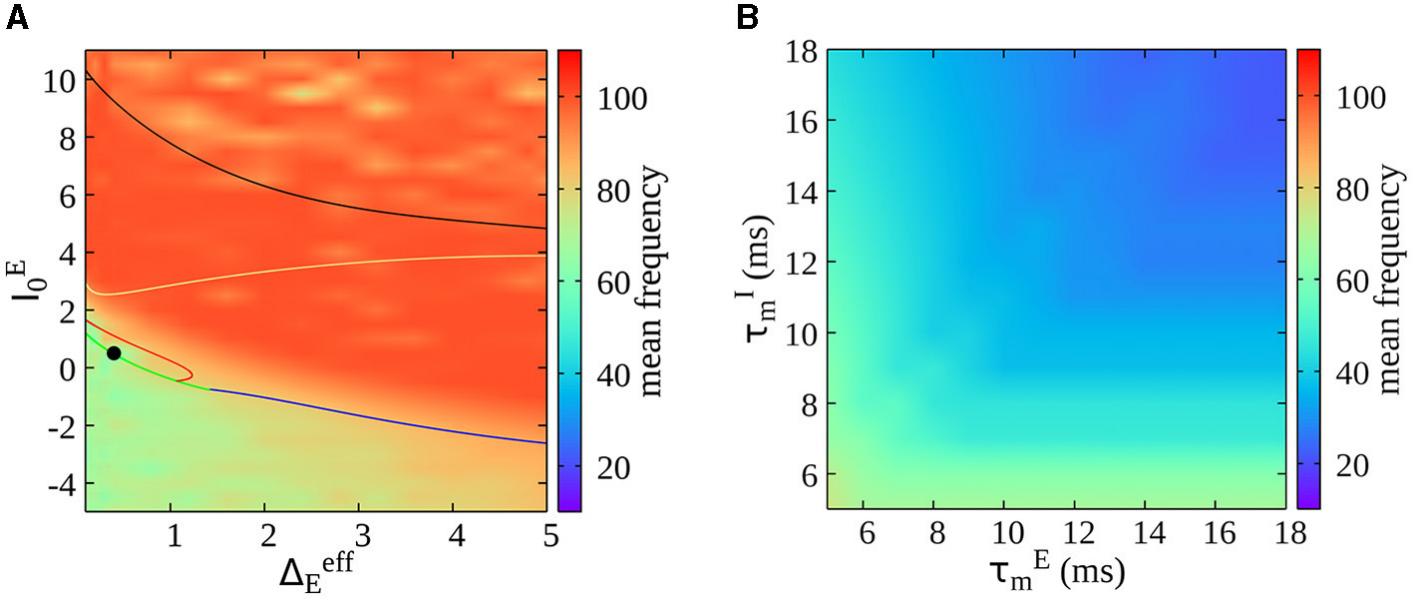
**(A)** Dependence of oscillations' frequency on external drive $I_{0}^{E}$ and neural heterogeneity ${\text{Δ}}_{E}^{eff}$. The frequency of oscillations is estimated as the mean of the power spectrum of the mean membrane potential of the excitatory population *V*^*E*^(*t*). This is a heat map of Figure 1A in the parameter space. **(B)** Oscillations' frequency as a function of the intrinsic membrane time constant of excitatory and inhibitory neurons ($\tau_{m}^{E}$ and $\tau_{m}^{I}$) within the bursting region [indicated by the solid black circle in **(A)**].

As a final result, we report here the dependence of oscillations' frequency on the model's parameters. We first estimate the frequency of oscillations (calculated as the mean of the power spectrum of the mean membrane potential of the excitatory population) as a function of the external drive and neural heterogeneity (see Figure 7A). Notice that a mean frequency of oscillations is also estimated in the asynchronous irregular region due to finite-size effects (refer to Figure 1A for the different regimes in the phase diagram). We observe that oscillations' frequency increases by increasing the mean drive to excitatory neurons ($I_{0}^{E}$), but it remains confined in the gamma range in the whole parameter space. In Figure 7B, we report instead the oscillations' frequency (for a value of $I_{0}^{E}$ and ${\text{Δ}}_{0}^{E}$ in the bursting region, see a solid black circle in Figure 7A) in function of the intrinsic membrane time constant of excitatory and inhibitory neurons. We observe a decrease in the oscillations' frequency for longer membrane time constants, showing that this model can be employed to study lower rhythms such as beta oscillations.

## 4 Conclusion

In this work, we have studied the emergence of bursting gamma oscillations in networks of spiking excitatory and inhibitory neurons. By employing an exact neural mass model, we could point to the different mechanisms responsible for different types of bursting oscillations. The first mechanism is due to finite-size fluctuations in spiking neural networks and appears in the vicinity of a bifurcation to oscillations in the neural mass models. We can call this mechanism Noise-induced Bursting Gamma oscillations (NiBG). While NiBG appears as deterministic in the large-dimensional spiking neural network, we needed to include explicitly additive noise in the low-dimensional neural mass model in the form of Gaussian noise to reproduce NiBG. Thanks to this approach, we could observe a good match between the NiBG observed in direct network simulations and those predicted by the neural mass model, with a good agreement in terms of their oscillations' frequency and their amplitude. While this approach was satisfactory for our case, the approximation of white noise for modeling finite-size fluctuations may be limited. Indeed, recent studies have confirmed that finite-size fluctuations have a non-trivial frequency spectrum (Klinshov and Kirillov, 2022). A possible future direction is to extend the theory by including a more refined model of finite-size fluctuations.

On top of the classical NiBG oscillations, we have proved the emergence of a new dynamical regime without noise sources in the neural mass model, called the IBG regime. Gamma bursts are deterministic emergent events due to collective chaos in the new IBG regime. Our model predicts that IBG and NiBG oscillations have different features in terms of the underlying structure of neurons' spiking activity. In the NiBG oscillations, all neurons have an irregular spiking activity (*CV*>0) that does not display a clear link with the ongoing gamma oscillation cycle. This is expected from a mechanism based on random fluctuations due to finite-size effects or external noise. Instead, in the IBG regime, there is a precise structure of neurons' spiking activity linked to the gamma cycle. In particular, the model shows that gamma bursts are related to a subgroup of bursting neurons in the network with high *CV* ~ 2. This prediction is consistent with recent experimental studies showing the crucial impact of bursting neurons on the emergence of gamma oscillations (Onorato et al., 2020).

Finally, we have demonstrated that the IBG regime is characterized by a higher capacity to interact with slower brain rhythms. We find that the network has a larger PAC to slower theta oscillations in the vicinity of the transition to IBG with respect to the region close to the transition to classical PING oscillations (NiBG regime). This depicts that the mechanism of IBG oscillations is a better candidate to optimally transfer information between brain regions than the classical NiBG oscillations.

Interestingly, we have shown that these results are quite general and can also be observed in sparse random networks. Guided by the neural mass model, we have performed numerical simulations in sparse networks, showing that the IBG can be observed in sparse networks as well and that it is characterized by a much larger PAC with theta oscillations with respect to classical PING limit cycles.

In the same direction, recent studies have shown a clear laminar organization of oscillatory components in Local Field Potentials. In particular, it is found there is a deep-to-superficial layer gradient of high-frequency power in cortical layers (Mendoza-Halliday et al., 2024). Our model demonstrates that it is possible to pass from gamma to beta bursts, changing inhibitory neurons' time constants (see Figure 7B). This is interesting because it is known that different interneurons have different densities along the laminar structure. For example, slower somatostatin interneurons are more prominent in deeper layers in contrast with fast parvalbumin interneurons (Tremblay et al., 2016). Future studies could employ the model here presented to test these predictions by specifically modeling fast and slow interneurons across cortical layers.

In this study, we have considered narrow-band gamma oscillations, but it is known that a second type of high-gamma oscillations (from 60 to 150 Hz) is broadband rather than purely oscillatory (Brovelli et al., 2005; Crone et al., 2006; Jerbi et al., 2009). We believe that this type of broadband oscillation could be emerging via a different mechanism, including spatially extended regions with gradients of model parameters. Including spatial structure and heterogeneity of parameters' values in this model is an interesting direction to possibly disentangle different types of gamma oscillations.

While the deterministic neural mass model correctly reproduces these gamma bursts, several steps should be taken to improve the model. Indeed, recent studies have shown that hippocampal gamma bursts appear at different frequencies (Douchamps et al., 2024). Our model cannot reproduce such variability completely, even if some variability is present across bursts. The origin of such a variety of gamma bursts is a very interesting direction that probably requires including a more realistic structure of networks' connections. Moreover, a more realistic model, e.g., including adaptation in pyramidal cells or the dynamics of synaptic receptors (Ferrara et al., 2023; Sheheitli and Jirsa, 2023), could be a good candidate to observe more complex spatio-temporal patterns as in experimental recordings (Douchamps et al., 2024).

Limits of the model: Our model relies on several assumptions that are important to be aware of for the interpretation of the results. First, the neural mass model is exact only when we consider a Cauchy distribution of neuron excitabilities. Several studies have shown that employing a Gaussian distribution of excitabilities (or of external noise) can lead to different emergent phenomena (Goldobin et al., 2021; Pyragas and Pyragas, 2022). While it is promising that the phenomenon of increased PAC for the IBG are maintained in the sparse network case, future studies should address the robustness of this phenomenon to different distributions of heterogeneities. Second, the neural mass model is exact only in the globally coupled case. While this is an unrealistic scenario, we have proved that the main results of this study (i.e., an increased PAC in the bursting gamma regime) stay valid for sparse networks with sufficiently large in-degree *K* (*K*>10). Third, the neural mass model is valid only for the quadratic-integrate and fire models; future studies could be developed through numerical simulations of neural networks to see whether the IBG appears also in other neural models.

## Data Availability

The raw data supporting the conclusions of this article will be made available by the authors, without undue reservation.
